# PerSurge (NOA-30) phase II trial of perampanel treatment around surgery in patients with progressive glioblastoma

**DOI:** 10.1186/s12885-024-11846-1

**Published:** 2024-01-26

**Authors:** Sophie Heuer, Ina Burghaus, Maria Gose, Tobias Kessler, Felix Sahm, Philipp Vollmuth, Varun Venkataramani, Dirk Hoffmann, Matthias Schlesner, Miriam Ratliff, Carsten Hopf, Ulrich Herrlinger, Franz Ricklefs, Martin Bendszus, Sandro M. Krieg, Antje Wick, Wolfgang Wick, Frank Winkler

**Affiliations:** 1 Neurology Clinic and National Center for Tumor Diseases, University Hospital Heidelberg, INF 400, 69120 Heidelberg, Germany; 2 Clinical Cooperation Unit Neurooncology, German Cancer Consortium (DKTK), German Cancer Research Center (DKFZ), 69120 Heidelberg, Germany; 3 Coordination Centre for Clinical Trials (KKS) Heidelberg, 69120 Heidelberg, Germany; 4 Department of Neuropathology, University Hospital Heidelberg, INF 224, 69120 Heidelberg, Germany; 5 CCU Neuropathology, German Consortium for Translational Cancer Research (DKTK), Geman Cancer Research Center (DKFZ), Heidelberg, Germany; 6 Department of Neuroradiology, University Hospital Heidelberg, INF 400, 69120 Heidelberg, Germany; 7 Department of Functional Neuroanatomy, Institute for Anatomy and Cell Biology, Heidelberg University, Heidelberg, Germany; 8 German Cancer Research Center (DKFZ), 69120 Heidelberg, Germany; 9 Biomedical Informatics, Data Mining and Data Analytics, University of Augsburg, Augsburg, Germany; 10 Neurosurgery Clinic, University Hospital Mannheim, 68167 Mannheim, Germany; 11 Center for Mass Spectrometry and Optical Spectroscopy (CeMOS), Mannheim University of Applied Sciences, Paul-Wittsack Str. 10, 68163 Mannheim, Germany; 12 Medical Faculty, Heidelberg University, Heidelberg, Germany; 13 Mannheim Center for Translational Neuroscience (MCTN), Medical Faculty Mannheim, Heidelberg University, Heidelberg, Germany; 14 Division of Clinical Neurooncology, Department of Neurology and Centre of Integrated Oncology, University Hospital Bonn, Bonn, Germany; 15 Department of Neurosurgery, University Hospital Eppendorf, Martinistr. 52, 20246 Hamburg, Germany; 16 Department of Neurosurgery, University Hospital Heidelberg, Heidelberg, Germany

**Keywords:** Glioblastoma, Recurrence, Cancer Neuroscience, Perampanel, AMPA receptor, Connectivity signature, Neuron-glioma synapse

## Abstract

**Background:**

Glioblastoma is the most frequent and a particularly malignant primary brain tumor with no efficacy-proven standard therapy for recurrence. It has recently been discovered that excitatory synapses of the AMPA-receptor subtype form between non-malignant brain neurons and tumor cells. This neuron-tumor network connectivity contributed to glioma progression and could be efficiently targeted with the EMA/FDA approved antiepileptic AMPA receptor inhibitor perampanel in preclinical studies. The PerSurge trial was designed to test the clinical potential of perampanel to reduce tumor cell network connectivity and tumor growth with an extended window-of-opportunity concept.

**Methods:**

PerSurge is a phase IIa clinical and translational treatment study around surgical resection of progressive or recurrent glioblastoma. In this multicenter, 2-arm parallel-group, double-blind superiority trial, patients are 1:1 randomized to either receive placebo or perampanel (*n* = 66 in total). It consists of a treatment and observation period of 60 days per patient, starting 30 days before a planned surgical resection, which itself is not part of the study interventions. Only patients with an expected safe waiting interval are included, and a safety MRI is performed. Tumor cell network connectivity from resected tumor tissue on single cell transcriptome level as well as AI-based assessment of tumor growth dynamics in T2/FLAIR MRI scans before resection will be analyzed as the co-primary endpoints. Secondary endpoints will include further imaging parameters such as pre- and postsurgical contrast enhanced MRI scans, postsurgical T2/FLAIR MRI scans, quality of life, cognitive testing, overall and progression-free survival as well as frequency of epileptic seizures. Further translational research will focus on additional biological aspects of neuron-tumor connectivity.

**Discussion:**

This trial is set up to assess first indications of clinical efficacy and tolerability of perampanel in recurrent glioblastoma, a repurposed drug which inhibits neuron-glioma synapses and thereby glioblastoma growth in preclinical models. If perampanel proved to be successful in the clinical setting, it would provide the first evidence that interference with neuron-cancer interactions may indeed lead to a benefit for patients, which would lay the foundation for a larger confirmatory trial in the future.

**Trial registration:**

EU-CT number: 2023-503938-52-00 30.11.2023.

## Background

With 3000 new cases per year in Germany, glioblastoma is the most common and most aggressive primary brain tumor. Its treatment is limited to surgery, radiotherapy, and chemotherapy, with the option of adding tumor-treating fields [1]. Newer, targeted therapies have not proven effective for the general patient population [2]. Glioblastoma show strong mechanisms of resistance, and thus after primary treatment, recurrence is inevitable. Recurrence is currently measured with primarily imaging-based criteria according to the response assessment in Neuro-oncology criteria (RANO); those MRI measurements are however not highly accurate and standardised [3].

Upon the recurrence of glioblastoma, there are no further standard treatment options [4] and the prognosis is poor (mean progression-free survival (PFS) 1.5 to 4.2 months and overall survival (OS) 8.6 to 9.1 months) [5]. Depending on the location, tumor burden, functional status and preconditions, a re-resection of the tumor can be beneficial for patients with recurrent glioblastoma and is currently performed in about a third of patients with recurrent glioblastoma [6, 7]. Since treatment options are limited at the point of recurrence, inclusion into a study is recommended for glioblastoma patients by the Society for Neuro-Oncology (SNO) and European Association for Neuro-Oncology [4, 8]. To develop further trials for patients, new biological insights of glioblastoma need to be translated from bench to bedside.

In recent years, a new concept of glioblastoma building an anatomical and communicating network among tumor cells and between glioblastoma cells and non-malignant brain cells such as neurons has emerged [9–12]. The network integration between glioblastoma cells is mediated by cellular membrane protrusions termed tumor microtubes (TMs, [9]). Through TMs, the glioblastoma cell network facilitates the tumor’s resistance to all standard therapies with highly connected glioblastoma cells surviving treatment and building more TMs to interconnect glioblastoma cells even more densely as well as mediating self-repair while non-connected cells die under treatment [9, 13].

Glioblastoma consist of highly invasive and proliferating cells populating the entire brain and a densely interconnected tumor core [9, 12, 14–16]. Both the invasive and more solid parts were found to be stimulated by synaptic input of neurons on glioblastoma cells [10, 11, 16]. Neurons build bona-fide synapses with glioblastoma cells, where glioblastoma cells are the postsynaptic partner. They receive growth and invasion stimuli from presynaptic neurons by postsynaptic α-amino-3-hydroxy-5-methyl-4-isoxazolepropionic acid (AMPA) receptors, preferentially located on TMs of glioblastoma cells [10, 11]. The inhibition of AMPA receptor activity by genetic or pharmacological perturbation with perampanel was shown to reduce glioma cell proliferation and invasion in patient-derived xenograft mouse models [10, 11]. Furthermore, with the preferential location of neuron-glioma synapses on TMs, they drive their formation, elongation, and dynamics, which are essential for the brain colonization of glioblastoma cells. Hence, the inhibition of AMPA-receptors by perampanel also reduced TM formation [17].

Because it is currently not known if perampanel shows comparable effects through inhibition of the neuron-glioma synapses in the human disease and if it could reduce tumor proliferation and potentially whole brain dissemination, this trial was developed to test perampanel in human glioblastoma.

The non-competitive AMPA-receptor inhibitor perampanel is an EMA-/FDA-approved compound for add-on therapy in epilepsy [18] with blood-brain-barrier penetrance and advantageous pharmacokinetics such as a terminal half-life 105 h and an effective half-life 48 h when no enzyme-inducing antiepileptic drugs are co-administered [19, 20]. For this indication it shows good efficacy by reduction of seizure frequency [21–23] and the tolerability in patients was satisfactory.

Up to half of all glioblastoma patients suffer from brain tumor-related epilepsy (BTRE) [24], with significant negative impact on quality of life. Perampanel has also been investigated in the treatment of BTRE, where it led to significantly improved seizure control in observational studies with a good tolerability profile [25–27]. Even in lower doses of 2–4 mg perampanel/day, studies have shown high responder rates of > 75%, and even no further epileptic seizures under treatment up to 50% [25–29].

Perampanel’s inhibitory effects on AMPA receptors on glioblastoma resulting in reduced tumor invasion, proliferation, and resistance-mediating formation of TMs in preclinical models paired with data for good tolerability and clinical applicability in patients lead to the PerSurge trial. Current data support a potential dual anti-tumor and anti-epileptic activity of perampanel in patients. In this proof-of-principle trial, the novel concept of disconnecting neuron-tumor networks is translated into the clinical practice. If the PerSurge trial provides a positive result, perampanel would become a candidate for a new tumor-specific therapy principle in glioblastoma.

## Methods and design

### Trial design

The PerSurge trial includes patients with recurrent or progressive glioblastoma with the indication for re-resection. At this stage of the disease, no established, efficacy-proven standard of care therapy is available. Patients will be carefully selected for the possibility to safely perform surgery after thirty days of perampanel treatment vs. placebo with the addition of an intermediate MRI to verify safety of postponing the surgery for a brief period. This is the basis for this window of opportunity trial concept where perampanel or placebo is applied and imaging-based readouts but also molecular analyses from resected material can be performed. While the resection itself is not part of the trial, its indication and the feasibility of its short delay are necessary for the inclusion into the PerSurge trial. The investigation of the resected tissue primarily by single nucleus RNA-Sequencing is crucial for verification of on-target effectivity of perampanel at the molecular level with confirmation of the new therapeutic concept to reduce glioblastoma connectivity by measuring the connectivity score [30]. The placebo group is deemed crucial to avoid any bias in analysis of the primary and secondary endpoints and the associated translational research.

Further continuation of perampanel or placebo after the surgery for another 30 days (treatment cycle 2) will not interfere with other tumor-specific therapies that might be planned after re-resection for the trial participant since wound healing after surgery and planning of the subsequent treatment is usually taking 4–5 weeks [8, 31]. Thus, after the postsurgical 30-day treatment cycle when the trial medication is concluded, the patient is able to timely start the next tumor-specific treatment such as re-radiation or chemotherapy, if medically indicated.

### Objectives and endpoints

#### Primary objectives and endpoints

The primary aim of the study is to evaluate changes in neuron-tumor synaptic connectivity in glioblastoma tissue and changes of tumor growth rate after 30-day perampanel treatment before surgery in comparison to placebo control. This will be assessed on the level of gene expression pattern levels evaluating the connectivity score from single cell RNA sequencing of resected tumor tissue [30]. Additionally, the tumor growth rate, i.e. the absolute change in log-transformed tumor volume [cm³], will be determined in blinded independent review of AI-supported quantifications of T2/FLAIR MRI studies between baseline and a pre-surgical MRI at day 30 after randomization [32]. Either objective will be regarded sufficient to prove effectivity of the treatment.

#### Secondary objectives and endpoints

Secondary objectives to evaluate the efficacy of perampanel in recurrent glioblastoma are the assessments of the kinetics in contrast-enhancing (T1CE images) tumor volume by central AI-based MRI analyses at the pre-surgical and postsurgical time point (day 30 and 60 after randomization) and T2/FLAIR tumor volume by central AI-based MRI assessment at the postsurgical time point. Especially the pre-surgical and post-surgical log-transformed tumor volume [cm³] per the Blinded Independent Review Committee (BIRC) are of interest. The health-related quality of life (HRQoL) and symptoms will be monitored by the patient questionnaires EORTC QLQ-C30 and QLQ-BN20 [33] and cognitive function will be surveilled with the mini-mental state examination (MMSE) [34]. The absolute change from baseline at post-operative day 30 in the C30 summary score and in total MMSE score will be evaluated. Overall survival (OS, time from the date of randomization to the date of death due to any cause) and progression-free survival (PFS, time from randomization until progression according to RANO criteria or death) further pose as secondary objectives [34]. Finally, a clinical evaluation of epileptic seizure activity by documentation of seizures by the patient will be performed at trial visits and the total number of epileptic seizures between baseline and post-operative day 30 (day 60 after randomization) will be analyzed. Safety will be evaluated by observing adverse events (AE), serious AEs and Suspected Unexpected Serious Adverse Reactions (SUSARs) of the trial drug until three days after the end of treatment.

#### Study duration and trial sites

The total duration of the recruitment for patients for this study is planned for 20 months (24 months first patient in– last patient out). It is planned to start in Q4/2023. Patients will be treated for 60 days with perampanel vs. placebo (two 30-day cycles) and followed-up after four weeks after the end of treatment for a safety visit. Further follow-up of OS and PFS will be examined every three months until death or last documented contact of the last randomized patient. At trial initiation, recruitment in 13 trial sites which are all large brain tumor centers in Germany is planned.

#### Trial population (incl. gender and age selection)

Patients with first or second recurrence of glioblastoma, the indication for re-resection of the recurrent tumor, and fulfillment of the other inclusion criteria will be eligible to participate in the trail. Inclusion of patients of an age of minimum 18 years will be irrespective of gender or ethnicity.

#### Inclusion criteria

1. Histologically confirmed glioblastoma, progressive or recurrent after 1 or 2 lines of prior treatment, involving one radiotherapy and drug treatments (temozolomide, lomustine, and/or other) or prior trial participation, > 3 months after end of radiotherapy, and therapy for relapse not yet started.
2. Indication for surgical resection of progressive or recurrent tumor tissue.
3. A sufficient amount of resected tumor tissue (minimum 0.5 cm^3^) is expected to be available for the trial-specific molecular, morphological, functional, and perampanel level analysis.
4. Tumor progression according to RANO criteria.
5. Age: ≥ 18 years.
6. Karnofsky Performance status score (KPS) ≥ 60% [35].
7. Life expectancy > 3 months.
8. Willing and able to comply with regular neurocognitive and health-related quality of life tests/questionnaires.
9. Written informed consent.
10. Cognitive state to understand rationale, necessity and individual consequences of study therapy and procedures.
11. Female patients with reproductive potential must use an approved contraceptive method during and for 4 weeks after the end of trial medication (Pearl Index < 1%).
12. Female patients with reproductive potential: a negative serum pregnancy test (beta-HCG) must be obtained prior to treatment start.

#### Exclusion criteria

1. Participation in other ongoing interventional clinical trials.
2. Inability to undergo contrast-enhanced MRI.
3. Inability to undergo surgery (e.g. because of need for continuous anticoagulation, known bleeding disorders, thrombocytopenia < 50/nl, preexisting wound healing problems).
4. According to the assessment of the local investigator, a safe waiting interval of 4–5 weeks for surgical resection is not possible because the growth dynamics, configuration, or location of the brain tumor, or any complication, require immediate or earlier surgical intervention to save the patient from harm (e.g. by herniation or other emergency situations, or brain damage due to tumor mass effects).
5. Any continued or planned standard or experimental treatment for the tumor other than resection, including antiangiogenic therapy (such as Bevacizumab), and local therapy in addition to the planned resection, including BCNU wafers, loco-regional hyperthermia, tumor bed irradiation, and photodynamic therapy.
6. Tumor carries a known mutation in the IDH1 or IDH2 gene.
7. Severe or significant abnormal (≥ Grade 3 Common Terminology Criteria for Adverse Events (CTCAE v5.0) [36] laboratory values for hematology (hemoglobin, white blood cells, neutrophils, or platelets), liver (serum bilirubin, ALT, or AST) or renal function (serum creatinine).
8. Known active tuberculosis; HIV infection or active Hepatitis B (HBV) or Hepatitis C (HCV) infection, or active infections requiring oral or intravenous antibiotics or that can cause a severe disease and pose a severe danger to lab personnel working on patients’ blood or tissue (e.g. rabies).
9. Any prior treatment with perampanel.
10. Pre-existing conditions like psychosis, aggression or suicidal thoughts that are considered as not allowing Perampanel treatment according to the assessment of the local investigator.
11. Concomitant intake of enzyme-inducing antiepileptic drugs (EIAEDs: carbamazepine, eslicarbazepine, oxcarbazepine, phenobarbital, phenytoin, primidon, rufinamid).
12. Steroid intake of more than 4 mg dexamethasone (or equivalence dose) in the last week, or expected indication for it in the foreseeable future.
13. History of other malignancies (except for adequately treated basal or squamous cell carcinoma or carcinoma in situ) within the last 2 years unless the patient has been disease-free for 2 years.
14. Any clinically significant concomitant disease or condition that could interfere with, or for which the treatment might interfere with, the conduct of the study or the absorption of oral medications or that would, in the opinion of the Principal Investigator, pose an unacceptable risk to the patient in this study.
15. Any psychological, familial, sociological, or geographical condition potentially hampering compliance with the study protocol requirements and/or follow-up procedures; those conditions should be discussed with the patient before trial entry.
16. Pregnancy or breastfeeding.
17. History of hypersensitivity to the investigational medicinal product or to any drug with similar chemical structure or to any excipient present in the pharmaceutical form of the investigational medicinal product.
18. The presence of any other concomitant severe, progressive, or uncontrolled renal, hepatic, hematological, endocrine, pulmonary, cardiac, or psychiatric disease, or signs or symptoms thereof, that may affect the subject’s participation in the study, according to investigators judgement.

### Interventions (Fig. 1; Table 1)

**Fig. 1:**
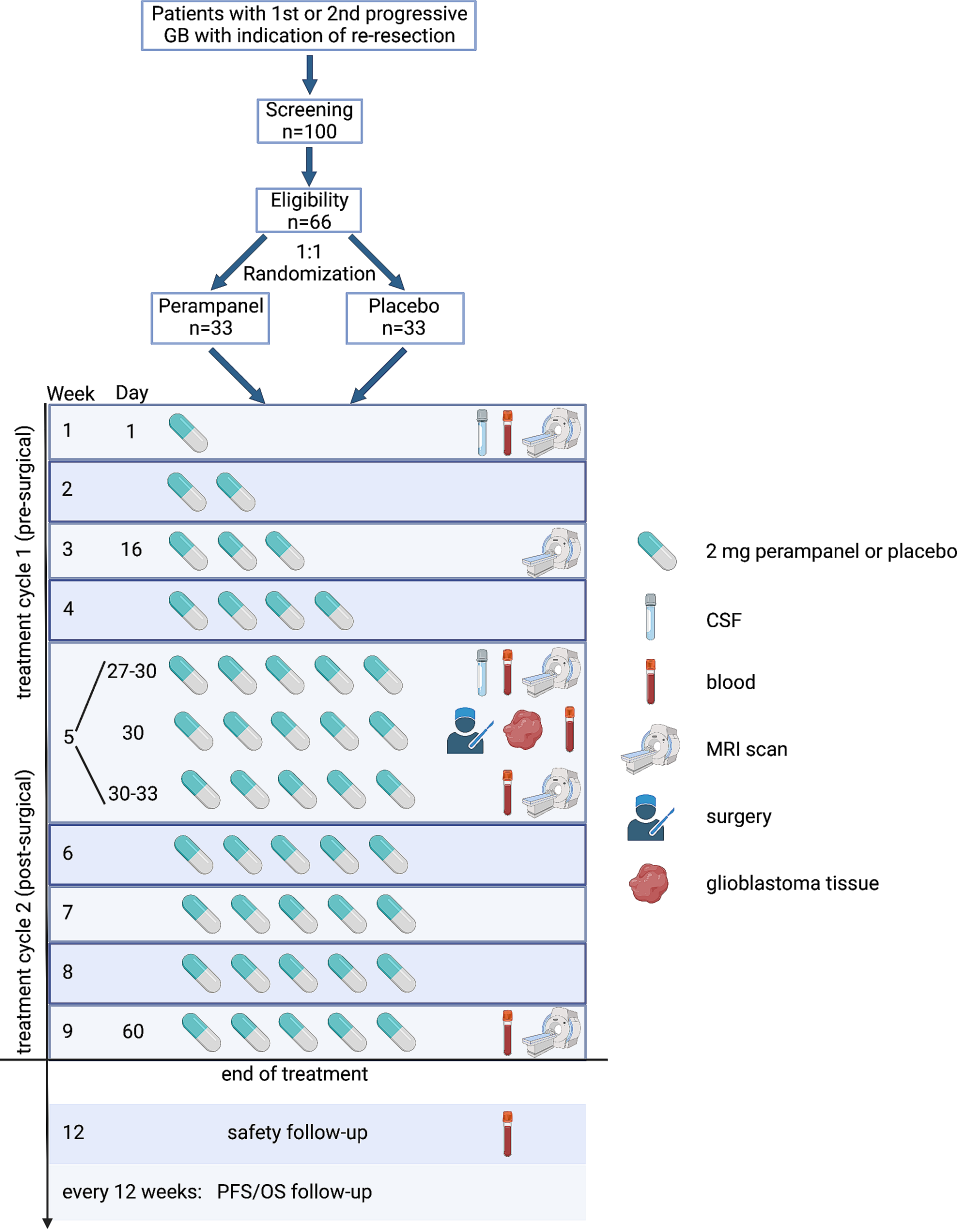
Clinical trial concept. Schematic overview of the trial. Created with BioRender.com

**Table 1:** Overview of visits and interventions

Study period	Enrolment	Treatment period (60 ± 3 days)					Post-treatment follow-up	
	Screening/Baseline						Safety follow up	OS & PFS follow-up
Visit no.	1	2	3	4	5	6	7	FU
**Timeline/Timepoint**	**D0**	**D16** **(± 2)**	**0–3 days** **prior** **resection**	**D30** **(± 3)** **tumour resection**	**0–3** **days** **after resection**	**POD30** **(± 3)** **EOT visit**	**4 weeks** **after last dose** **(EOT visit)**	**Every** **3 months**
Mode of scheduled visit	Outpatient/inpatient	Outpatient/inpatient	Outpatient/inpatient	Inpatient	Outpatient/inpatient	Outpatient/inpatient	Outpatient/inpatient	Outpatient/ tel. visit
Written Informed Consent^a^	**X**							
In-/Exclusion Criteria	**X**							
Demographics, medical and surgical history/ preexisting conditions (evaluated in analogy to CTCAE)	**X**							
Randomisation*	**X**							
Vital signs^b^	**X**		**X**			**X**	**X**	
Physical and neurological examination	**X**	**X**	**X**		**X**	**X**	**X**	
Concomitant medications/procedures	**X**	**X**	**X**	**X**	**X**	**X**	**X**	**X** ^c^
Karnofsky Performance Status Scale	**X**	**X**	**X**		**X**	**X**	**X**	
Mini Mental State Examination	**X**					**X**		
HRQoL: Patient questionnaires EORTC QLQ-C30, QLQ-BN20	**X**					**X**		
Adverse Events		**X**	**X**	**X**	**X**	**X**	**X**	
Documentation of epileptic seizures	**X** ^d^	**X**	**X**		**X**	**X**	**X**	
***Intervention***								
Dispensing of trial drug^e^	**X**	**X**	**X**		**X**			
Return of trial drug		**X**	**X**		**X**	**X**		
Study drug compliance check^f^		**X**	**X**	**X**	**X**	**X**		
***Tumour (MRI) and response assessment***								
**Gd-MRI** (volumetric assessment of tumour sample)^g^	**X**	**X**	**X**		**X**	**X**		
Progression (RANO criteria, imaging-based)		**X**	**X**		**X**	**X**	**X** (if MRI performed, according to local standards)	**X** ^h^
Survival status		**X**	**X**	**X**	**X**	**X**	**X**	**X** ^h^
***Laboratory evaluations***								
Haematology	**X** ^i^		**X**		**X**		**X**	
Clinical chemistry	**X** ^i^		**X**		**X**		**X**	
Pregnancy test	**X**		**X**			**X**		
Asservation of tumour material^j^, **assessment of connectivity score**				**X**				
Serum perampanel concentration^k,+^				**X**				
Serum for exploratory biomarkers for network connectivity ^+^	**X**			**X**		**X**		
Cerebrospinal fluid (CSF)^l,+^	**X**			**X**				
Plasma (Determination of extracellular vesicles)^+^				**X**				

Bold font means assessment for derivation of one of the two primary efficacy outcomes. Tumour resection is no trial-specific procedure and no component of the intervention

*All baseline characteristics (including vital signs, laboratory data, medical history, prior medication,…) must be assessed before randomisation

^+^Exploratory/Translational research (not part of the main trial)

^a^Written informed consent can be obtained up to 7 days prior to randomisation, but before any study-specific intervention/assessment

^b^Vital signs: blood pressure, pulse rate, temperature

^c^In long-term FU only subsequent anticancer therapies will be documented

^d^Based on the last 4 weeks

^e^Experimental/ control intervention: capsules once daily before going to bed (weekly increases up to 1 × 5 capsules / day)

^f^Treatment adherence (check of patient’s dosing diary and the counting of the number of returned capsules) at every study visit

^g^Gadolinium Magnetic Resonance Imaging; Day 0 (-4 days): reference MRI; Day 16 (± 2 days): “safety MRI” to detect patients who need earlier resection (before day 30 post-randomisation) according to the assessment of the local investigator

^h^Progression determined by the investigator according to RANO without central review [AI-based]) by the BIRC, and survival status during long-term follow-up will be determined for every patient by phone interviews or by e-mail or regular patient follow-up documentations at the respective site every 3 months until last patient out

^i^Up to 7 days prior to randomisation as part of routine laboratory analyses

^j^Relapse tumour resection 30 [28–34] days after Day 0. Tissue asservation should take place 10–18 h after last study drug intake: [1] fresh frozen, [2] FFPE, [3] 4% PFA; for measurement of perampanel tissue levels and study drug-dependent tumour tissue effects. On the day prior to surgical tumour removal, the exact time of intake of trial medication has to be documented

^k^Serum sample (Perampanel) is taken 10–14 h after last study drug intake

^l^At baseline, CSF is collected by lumbar puncture in a suitable plastic tube. On the day of surgery, CSF can be collected intraoperatively or by lumbar puncture

#### Treatment regimen and dosing

**Fig. 2:**
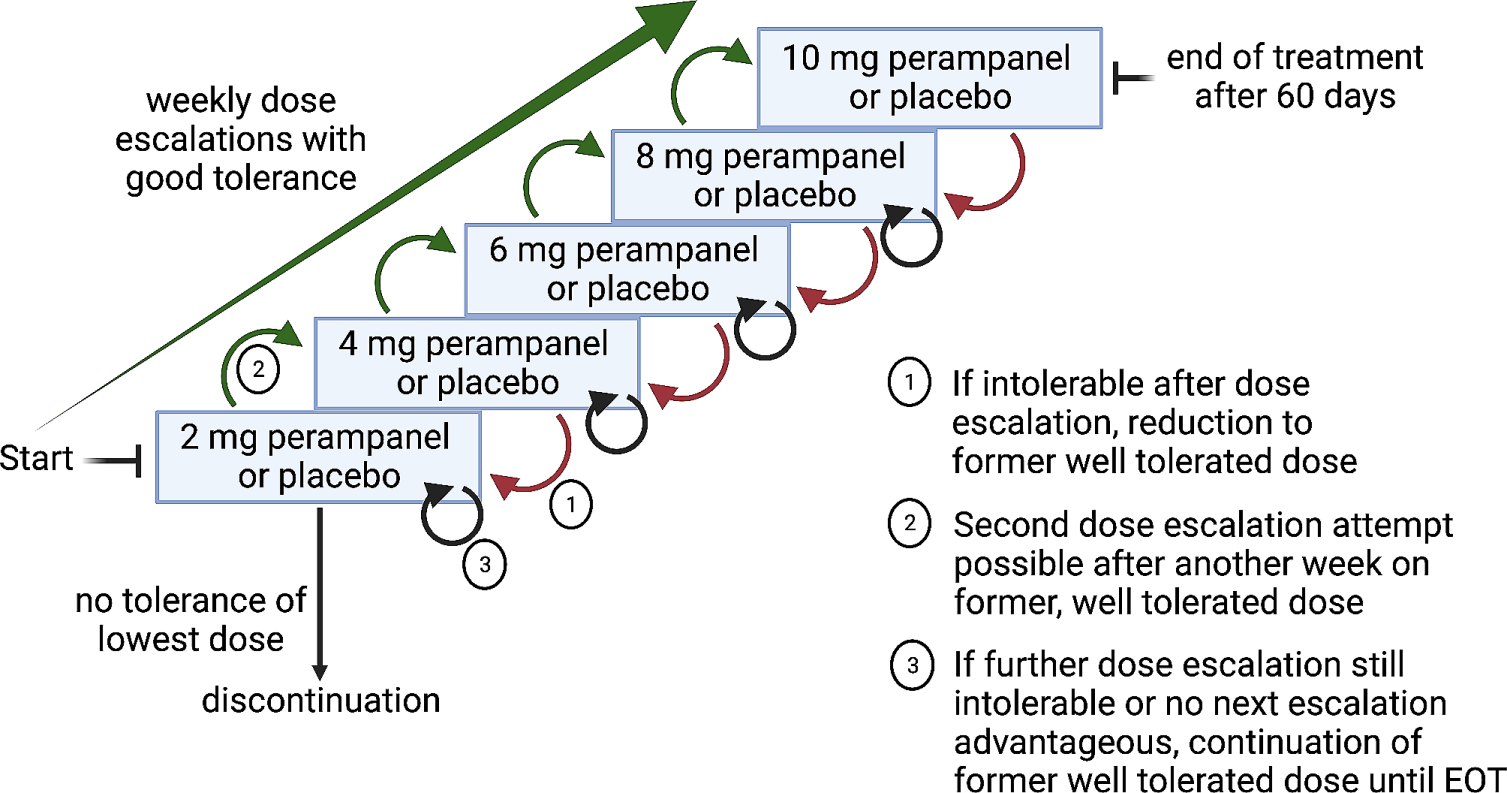
Dose escalation workflow of the study drug. Illustration of the dose escalation concept of the trial. Created with BioRender.com

After 1:1 randomization of perampanel vs. placebo, treatment will be started at day 1 with one capsule of 2 mg perampanel at night before bedtime vs. one capsule placebo. Provided good tolerability, the dose will be increased by one capsule of 2 mg perampanel or one capsule placebo at night per week up to 10 mg/day which equals to five capsules perampanel or placebo per night (Fig. 2). If tolerated well, this dosing will be continued until the end of the study. The total duration of the drug application is 60 days (57–63 days) in two cycles (30 days pre- and 30 days post-operative). The escalation scheme is in line with the regular dose escalation regimen of perampanel if applied in epilepsy [21]. If side effects such as drowsiness, dizziness, nausea, confusion, irritability, gait instability, coordination or mental changes occur after dose escalation, the dose will be reduced to the previously well tolerated dose. Another attempt of increasing the dose can be made after a waiting period of seven days, or the lower, tolerable dose will be maintained until the end of the study. If the first dose level (1 capsule = 2 mg per day) proves intolerable, the intake of the drug is permanently ended. These evaluations of dose reductions will be done clinically by the investigator.

#### Imaging

During the study, five MRIs with gadolinium-based contrast enhancing agent will be performed to monitor tumor size. First, an MRI at the start of the study will be used as a baseline measurement of tumor volume. After randomization, an interim MRI at D16 (+/-2 days) will be assessed for safe continuation of the pre-surgical cycle up until day 30 versus the need to proceed with surgery earlier. The third MRI will be performed the 0–3 days prior to resection and the fourth MRI 0–3 days after resection. The last MRI will be performed at day 30 of the post-surgical cycle at the end of the study. The analysis will be performed with AI-based assessment of growth kinetics.

#### Blood sampling

Blood will be drawn six times during the study. Hematology and clinical chemistry will be assessed at baseline, 0–3 days before, and 0–3 days after resection as well as at the point of safety follow-up. Additional sampling for translational analyses of network connectivity markers will be acquired at baseline, the timepoint of surgery and at the end of the trial at post-operative day 30. Perampanel serum concentrations as well as plasma levels of extracellular vesicles in the plasma will be assessed from blood probes at the timepoint of surgery.

#### Cerebrospinal fluid sampling

Cerebrospinal fluid (CSF) will be obtained for translational research twice during the study, once at baseline, and secondly at the timepoint of surgery– either intraoperatively or via lumbar puncture.

#### Surgery and tumor tissue

While the surgical resection of the recurrent glioblastoma itself is not part of the study, the resected material will be analyzed for the primary objective of changes in neuron-tumor connectivity from single nuclei RNA sequencing as well as translational research, including measurement of perampanel tumor tissue concentrations.

### Adverse events

Adverse events (AE) are defined as any untoward medical occurrence in a patient who the study medication is applied to and that is not necessarily caused by the investigational medicinal product. AEs will be documented and graded by CTCAE V 5.0 [36] throughout the trial. This includes clinically relevant or relevant worsening of baseline physical and neurological findings as well as vital signs, electrocardiographies, and laboratory results. AEs will be assessed for their seriousness, relationship with the intervention, outcome, and the need to take action (e.g. changes in dosing) by the investigator. Serious adverse events (SAEs) are defined as any untoward medical occurrence that results in death, is life-threatening, requires or prolongs hospitalization, leads to persisting significant disability, birth defect, or is otherwise medically relevant. SAEs have to be reported to the pharmacovigilance department in the coordination center for clinical trials in Heidelberg within 24 h of becoming known to the investigator. All AEs throughout the trial will be summarized and displayed.

### Statistical considerations

The statistical analysis will be performed by KKS (Coordination Center for Clinical Trials) Heidelberg. For the evaluation of the co-primary endpoints, the connectivity score and MRIs will be assessed. The connectivity score is calculated by subtraction of up- and downregulated connectivity score genes using bioinformatic analyses. The second primary endpoint of tumor growth rate in MRI from day 0 to day 30 will be measured with AI-based evaluation of log-transformed changes in tumor volumes over time using a linear mixed effects model.

Based on earlier studies [10, 30], it was determined that analysis of 25 patients from each group will lead to a 90% power to detect a probability of 0.211 that an observation is reduced in the perampanel group compared to the placebo group. A Wilcoxon (Mann-Whitney) rank-sum test with a 0.025 two-sided significance level will be applied. A normal distribution and effect size of 1.125, and more precisely, a minimum difference in means of 0.9 and a common standard deviation of 0.8, was presumed for the calculation of the probability of 0.211.

Earlier studies have proven high accuracy of longitudinal assessments of tumor burden [32] and a mean glioblastoma growth rate of 54% (+/- 15%) in 30 days [37]. Thus, based on expert estimation, a reduction of the tumor growth rate of 36% (+/-15%) within 30 days would be regarded as clinically relevant. Assuming normally distributed values, with a power of > 94% the above-mentioned difference in tumor growth rate could be detected if the sample size is 25 patients per group, using the Wilcoxon (Mann-Whitney) rank-sum test with a 0.025 two-sided significance level.

Thus, for statistical analyses, the study will include *n* = 50 patients to be analyzed, which was extrapolated to mean a screening for eligibility for *n* = 100 patients and allocation of *n* = 66 in a 1:1 randomization in each arm (perampanel vs. placebo) after assuming consent on 65% of eligible patients. It was calculated that including 25% missing information for the primary outcomes (no surgery performed, e.g. due to death before V2, i.e. connectivity score missing), 66 patients should be allocated to achieve 50 out of them to be analyzable. The secondary and translational outcomes will be analyzed exploratively.

#### Translational research

For further investigations, the acquired samples will be exploratively analyzed. Additional to the primary objective assessment, the resected tumor tissue will be explored on bulk RNA sequencing level, exome sequencing, DNA methylation analysis, proteomic readouts and stained for AMPA-receptors and TM-networks. Freshly resected tissue will also be used for calcium imaging and whole-cell patch clamping of glioblastoma cells to further investigate effects of the treatment on (inter)cellular communication [10, 11]. Finally, liquid chromatography - mass spectrometry (LC-MS) will be performed to measure perampanel tumor tissue levels.

CSF samples will also be evaluated for proteins involved in tumor cell connectivity [38]. Extracellular vesicles from blood plasma will be evaluated [39]. Perampanel serum and tumor tissue levels will be measured at the point of resection. All translational data will be integrated and a database built to multimodally dissect the specific AMPA-receptor inhibitory effects and potentially identify a biomarker for perampanel antitumor efficacy.

## Discussion

This study translates novel fundamental insights into glioblastoma as a disease driven by direct neuronal input, specifically neuron-glioma synapses that stimulate key features of malignancy, into a clinical trial concept where the AMPA receptor inhibitor perampanel is tested to inhibit tumor connectivity and growth. With the clear unmet clinical need to improve the basic understanding and subsequently the therapy of glioblastoma, it is necessary to translate new biological insights into clinical application, dissect disease subgroups, and monitor treatment effects on the target level in individual tumors. The objective to measure tumor connectivity based on single nuclei RNA sequencing further strengthens the goal to molecularly profile tumors for decision making and to establish molecular biomarkers for the monitoring of treatment efficacy with respect to the biological target. The planned acquisition of sample material in this study to test for tumor cell connectivity before and under treatment with further translational analyses regarding other molecular underpinnings as well as drug distribution in blood and tumor tissue will not only allow to directly link potential treatment benefits or challenges with biological changes but also establish at which time point and location benefits or the lack thereof can be observed. Importantly, the PerSurge trial allows to relate those biological effects to antitumor effects via exploitation of newly established AI-based MRI imaging readouts that shall allow to capture even short-term effects on tumor growth.

With the PerSurge trial, key new insights into glioblastoma biology, particularly the relevance of neuron-tumor and tumor-tumor networks, are further translated into clinical application. While this is the first investigation of selective AMPA-receptor inhibition in glioblastoma to reduce neuron-glioma synaptic communication, a first German national multicenter trial using meclofenamate (MFA) with temozolomide in recurrent glioblastoma is already applying the principle of a tumor-tumor disconnecting therapy, as the study drug inhibits gap junctions [40, 41]. Together this paves a new road for therapies specifically designed for network inhibition in glioblastoma. The primary and secondary endpoints and the extensive translational research will help to clarify whether those therapies are indeed employing their target in the human disease, whether this can lead to meaningful antitumor effects, and whether biomarkers can help to guide future patient selection. If successful, the PerSurge trial can provide valuable data for a future larger confirmatory trial of perampanel in glioblastoma.

## Data Availability

Translational research data will be available from the corresponding author upon reasonable request.

## Abbreviations

AE: adverse event
AI: artificial intelligence
AMPA: α-amino-3-hydroxy-5-methyl-4-isoxazolepropionic acid
BIRC: Blinded Independent Review Committee
BMBF: Bundesministerium für Bildung und Forschung, German Federal Ministry of Education and Research
BTRE: brain tumor-related epilepsy
CTCAE: Common Terminology Criteria of Adverse Events
EANO: European Association for Neuo-Oncology
EMA: European Medicines Agency
FDA: Food and Drug Administration
HRQoL: health-related quality of life
KPS: Karnofsky Performance Status
MFA: Meclofenamate
MMSE: Mini Mental State Examination
MRI: Magnetic resonance imaging
OS: Overall survival
PFS: Progression free survival
QoL: Quality of Life
RANO: Response Assessment in Neuro-Oncology Criteria
RNA: ribonucleic acid
SAE: Serious adverse event
SNO: Society for Neuro-Oncology
SUSAR: Suspected Unexpected Serious Adverse Reaction
